# Digital-Droplet PCR for Quantification of CD19-Directed CAR T-Cells

**DOI:** 10.3389/fmolb.2020.00084

**Published:** 2020-05-15

**Authors:** Thomas Mika, Abdelouahid Maghnouj, Susanne Klein-Scory, Swetlana Ladigan-Badura, Alexander Baraniskin, Julia Thomson, Justin Hasenkamp, Stephan A. Hahn, Gerald Wulf, Roland Schroers

**Affiliations:** ^1^Department of Medicine, Hematology and Oncology, Ruhr University Bochum, Bochum, Germany; ^2^Department of Molecular Gastroenterologic Oncology, Ruhr University Bochum, Bochum, Germany; ^3^IMBL, Universitätsklinikum Knappschaftskrankenhaus Bochum, Bochum, Germany; ^4^Clinic for Hematology and Medical Oncology, Georg-August University, Göttingen, Germany

**Keywords:** CD19-directed chimeric antigen receptor (CAR) T-cells, axicabtagene ciloleucel, aggressive lymphoma, digital-droplet PCR (ddPCR), flow cytometry (FCM), immunotherapy

## Abstract

CD19-directed CAR-T-cells (CD19-CAR) have demonstrated remarkable clinical results in patients suffering from refractory or relapsed lymphoma and acute lymphoblastic leukemia. In order to further optimize follow-up, to explain treatment failure, and to control adverse events biomarkers for monitoring of response are urgently needed. Peak expansion and persistence are correlated with response rates and severity of side effects. However, no standardized method or commercially assay for CD19-CAR measurement is established yet. In this study, two primer-probe assays for digital-droplet PCR (ddPCR) were designed and subsequently explored on 54 samples collected from seven patients after CD19-CAR treatment with axi-cel over time. Detection and quantification of CAR-T-cells were feasible and reliable for all patients included. Peak expansion measured with our assay significantly correlated with the grade of neurologic adverse events but not with cytokine release syndrome. All patients with loss of CAR-signal eventually had disease progression. In summary, our novel assay allows monitoring of CAR-T-cells *in vivo* and may add to safety and efficacy of CAR-T treatment.

## Introduction

Chimeric antigen receptor (CAR) cells represent an emerging treatment option with noteworthy activity in different tumor diseases. CAR cell therapy is based on genetic engineering of immunologic effector cells, such as T lymphocytes or natural killer (NK) cells, mostly by viral *in vitro* transduction. CARs comprise extracellular antigen-binding domains cloned as single chains from antibodies' variable regions, which are linked to intracellular signaling domains derived from human T-cell receptor and fused costimulatory molecules. CAR expression on T-cell or NK-cell surfaces enables specific recognition and killing of target tumor cells.

Currently, two CD19-directed CAR constructs transduced into T-lymphocytes, tisagenlecleucel and axicabtagene ciloleucel (axi-cel), are approved and commercially available for treatment of diffuse large B-cell lymphoma, primary mediastinal large B-cell lymphoma, and transformed follicular lymphoma. Also, tisagenlecleucel is available for therapy of B-lineage acute lymphoblastic leukemia. Despite significant clinical activity of both CAR-T-cell products in advanced disease and treatment stages, therapeutic failure is ultimately observed in 30–50% of patients harboring DLBCL (Locke et al., 2019). Loss of target (CD19) antigen and T-cell exhaustion are potential explanations. Yet, better understanding of mechanisms and advanced therapeutic monitoring of CAR-T cells are warranted to further optimize the efficacy and long-term success of CAR-T cell therapies.

Recently, *in vivo* CAR-T-cell expansion following re-transfusion has been recognized as important surrogate marker for lymphoma response and disease control (Neelapu et al., 2017; Park et al., 2018; Schuster et al., 2019). Similar observations have been made in CAR-T cell clinical approaches against chronic lymphatic leukemia and multiple myeloma (Fraietta et al., 2018; Cohen et al., 2019; Hirayama et al., 2019). Besides, CAR-T cell expansion is associated with adverse treatment events (Maude et al., 2014; Neelapu et al., 2017), and long-term CAR-T cell persistence in peripheral blood indicates enhanced anti-tumor efficacy (Milone et al., 2009). Together, these findings highlight the importance of accurate CAR-T-cell measurements in peripheral blood for clinical follow-up and scientific purposes. At this point, quantitative real-time PCR and flow cytometry (FCM) have been introduced for detection of CAR-T cells in peripheral blood samples. However, both techniques have inherent disadvantages limiting their application in clinical routine and research settings (Kochenderfer et al., 2012; Maude et al., 2014; Cohen et al., 2019).

Digital-droplet PCR (ddPCR) represents a modern technique with increasing application in laboratory diagnostics of hematologic disease. Previously, we explored a ddPCR approach for chimerism analyses following allogeneic hematopoietic stem cell transplantation. ddPCR compared favorably to qRT-PCR as to robustness and routine applicability in this setting (Mika et al., 2019).

In the present study, we developed a ddPCR assay for analysis of axi-cel in peripheral blood. We were able to reliably detect axi-cel in several blood samples collected from patients treated in two German treatment centers during a follow-up period of 6 months. The ddPCR assay gave reproducible CAR-T-cell quantifications, and, concordant results compared to parallel CAR-T cell enumeration by FCM could be demonstrated.

## Materials and Methods

### Patients and Sample Preparation

Blood samples were drawn from patients who had been treated with axicabtagene ciloleucel (axi-cel) at two German treatment centers (Department of Hematology and Oncology, Bochum and Department of Hematology and Oncology, Göttingen). All patients had measurable disease at the time of axi-cel treatment. Treatment was carried out according to manufacturer's instructions with lymphodepleting chemotherapy comprising fludarabine (30 mg/m^2^) and cyclophosphamide (500 mg/m^2^). Patients had given informed consent, and, the study was approved by the local Ethical Committee (#19-6750). Further patient information is given in Table 1.

**Table 1:** Patients characteristics and clinical outcome including therapies prior to axi-cel.

**Patient number**	**Age**	**Type of disease**	**Initial diagnosis**	**1st-line therapy**	**2nd-line therapy**	**3rd-line therapy**	**Apheresis/CAR-T application**	**CRS/ICANS**	**Follow-up**
1	60–64	DLBCL	01/18	6 x R-CHOP14 (PR)	1 x R-DHAP 1 x R-DHOx (PD)	–	Apheresis 05/19 Bridging: R-GemOxDex + RT Transfusion: 06/19	CRS: 1 ICANS: 2	3 months: PR 6 months: PR 9 months: PR
2	60–64	DLBCL	12/15	6 x R-CHOP14 + 2 x R + RT (CR)	3 x R-ICE (PR)	3 x R-ICE (PR)	Apheresis 05/19 Bridging: R-ICE Transfusion: 06/19	CRS: – ICANS: –	3 months: PD 6 months: Lost to follow-up
3	55–59	DLBCL	10/18	6 x R-CHOP14 (PD)	3 x R-DHAP (PD)	GM-ALL B-NHL (PD)	Apheresis 07/19 Bridging: R-ICE Transfusion: 09/19	CRS: – ICANS: –	3 months: SD 01/20: PD
4	65–69	Transformed FCL	06/04 FCL 03/18 transformation (DLBCL)	6 x R-CHOP14 + RT + Surgical Debulking^*^ (PD)	2 x R-DHAP (PD)	–	Apheresis 08/19 Bridging: none Transfusion 09/19	CRS: 2 ICANS: –	3 months: PR 6 months: PR
5	70–75	DLBCL	02/14	6 x R-CHOP14 + 2 x R (CR)	3 x R-DHAP + BEAM + auto-TX (CR)	RT	Apheresis 08/19 Bridging: none Transfusion: 09/19	CRS: 2 ICANS: 3	3 months: CR 6 months: CR
6	60–64	DLBCL	06/18	6 x R-CHOP14 + 2 x R (PR)	1 x R-DHAP Switch to 1 x R-ICE (PR)	Allo-Tx (PR)	Apheresis 01/20 Bridging: none	CRS: 1 ICANS: –	N/A
7	70–75	Transformed FCL	06/16 04/19 transformation	6 x R-CHOP14 + 2 x R (PR) + RT ^*^	2 x R-Gem-Ox (PD)	R-Polatuzumab (PD)	Apheresis: 01/20 Bridging: none	CRS: – ICANS: –	N/A

*Cytokine release syndrome (CRS) and immune effector cell-associated neurotoxic syndrome (ICANS) were graded according ASTCT guideline (Lee et al., 2019). Auto-Tx, autologous stem cell transplantation; Allo-Tx, allogeneic stem cell transplantation; PR, partial response; CR, complete response; PD, progressive disease; SD, stable disease*.

* *Treatment after lymphoma transformation*.

For all samples (positive controls, negative controls, and patients), DNA extracted from peripheral blood mononuclear cells was used. After collection of blood samples, PBMC were isolated by density gradient centrifugation. Genomic DNA (gDNA) extracted from residual cells in an axi-cel product bag using the QiaAMP Mini Kit (Qiagen™) advanced by RNAse digestion (Qiagen™) was used as a positive control. Parallel flow cytometry was performed in nine patient samples, where enough cells for gDNA isolation and flow cytometry were available. gDNA isolated from un-transduced PBMC was used as a negative control. B-cell counts were obtained retrospectively as part of routine follow-up tests in the centers' clinical laboratories.

### Design of Digital PCR Primers and Probes

Axi-cel specific DNA was amplified from gDNA by conventional PCR with primers binding to the long terminal repeats (LTR) of the retrovirus originally used for vector cloning (YESCARTA, 2018). The amplicon was purified from agarose gel (gel-extraction kit, Nippon Genetics) and sequenced. Primer sequences are given in Supplementary Figure 1A.

Subsequently, two primer-probe assays (*FMC63-28Z-1 and FMC63-28Z-2)* were designed with Primer 3 software and ordered from BioRad™. Primer-probe sequences are given in Supplementary Figure 1B. In each assay, both primers bind to the FMC63 derived sequence within the original construct.

### Digital PCR

Digital PCR was performed as previously described by our group (Mika et al., 2019). The reaction was set up in 20 μl sample volume containing 10 μl of 2 × ddPCR Supermix (no dUTP, BioRad™), 1 μl FAM-labeled FMC63 primer-probe-assay, 1 μl Hex-labeled housekeeper primer-probe assay, 0.4 μl EcoRI restriction enzyme (Fast digest, NEB™) and 10–75 ng gDNA (volume 5 μl), and nuclease-free water to adjust the sample to the final volume. HEX labeled assays (BioRad™) detecting reference genes *RPPH1* and *TERT* were used as control housekeeping genes (Supplementary Figure 2). The final sample was incubated for 10 min at 36°C for restriction digestion. Afterwards, droplets were generated in a QX200 droplet generator (BioRad™) according to manufacturers' instructions, giving a final volume of 40 μl.

DNA amplification was carried out using the following PCR program: initial denaturation at 95°C for 10 min, amplification with 40 cycles at 94°C for 30 s followed by 60°C for 1 min, and a final step at 98°C for 10 min (C1000 Touch Thermal Cycler, BioRad™). Ramp rate was set to 2.5°C/s. Finally, droplets were analyzed in a QX200 droplet reader (BioRad™), and, data was processed with QuantaSoft software (BioRad™) including Poisson's distribution analysis.

Based on random distribution of the target sequence into >10,000 droplets per well, Poisson's statistics enables absolute quantification of the target. The number of target copies per droplet was calculated based on the number of negative droplets in the sample. Since each droplet has a defined volume, calculation of copies/μl in the analyzed sample is possible (e.g., 10 target copies in 1,000 analyzed droplets, droplet volume = 1 nl−10 copies/1 μl). Data analysis resulted in absolute copies/μl of the target in the sample volume. Since the amount of DNA in each sample is versatile biased, fractional abundance (below) was used to describe relative increment or decline of CD19-CAR gene in relation to housekeeping genes. All samples were analyzed in two different assays (FAM-FMC63-28Z-1 + HEX-RPPH1, FAM-FMC63-28Z-2 + HEX-TERT) in technical duplicates, respectively. For determination of absolute copies/μl, mean fractional abundance of duplicates [FAM CD19 copies/μl: (FAM CD19 copies/ μl + Hex reference copies/μl)] was calculated (Supplementary Figure 3).

### Analytical Performance of the Assay

Reproducibility and precision were assessed by replicate tests of a positive control (product bag wash-out, 1 mean viral copy per genome, assessed by ddPCR) and negative control. Accuracy and limit of detection were determined by repeated measurements of dilution series of the positive control (each *n* = 5). Limit of blank (LOB) was calculated based on the replicate tests of negative control samples (Armbruster and Pry, 2008).

For spike-in experiments, mixed samples were prepared from positive control and negative control samples. DNA concentration per well was constant at 15 ng in a 20 μl reaction volume. Dilution series of mixed samples were prepared, with the lowest dilution comprising 2.1 ng positive control DNA spiked into 210 ng negative control DNA (10^−4^).

### Flow Cytometry (FCM)

The recently introduced CD19-CAR detection kit (Miltenyi Biotec™) was used to detect CAR-T-cells by FCM. In brief, anti-Biotin staining was performed with Biotin VioBright FITC antibody. Additional antibodies were used for CD3, CD4, and CD8 staining (Miltenyi Biotec™). Staining was carried out according to manufacturers' instructions. No live/dead staining was performed; however, gating was done by FSC/SSC evaluation and CD3 staining. FCM was performed on a BD FACS Canto II machine. Data were analyzed using FlowJo v.10.6.1 software. Fractions of CD3^+^/CAR^+^-cells were enumerated and correlated with fractional abundance as measured by ddPCR in the same sample.

### Statistical Analysis

Regression analysis (linear or log), Pearson correlation, and two-tailed *t*-tests were used for correlation analyses. All statistical analyses and data plots were carried out with GraphPad Prism software (Version 5).

## Results

### Amplification of CD19-CAR Gene From Recipient CAR-T Cells

**Figure 1:**
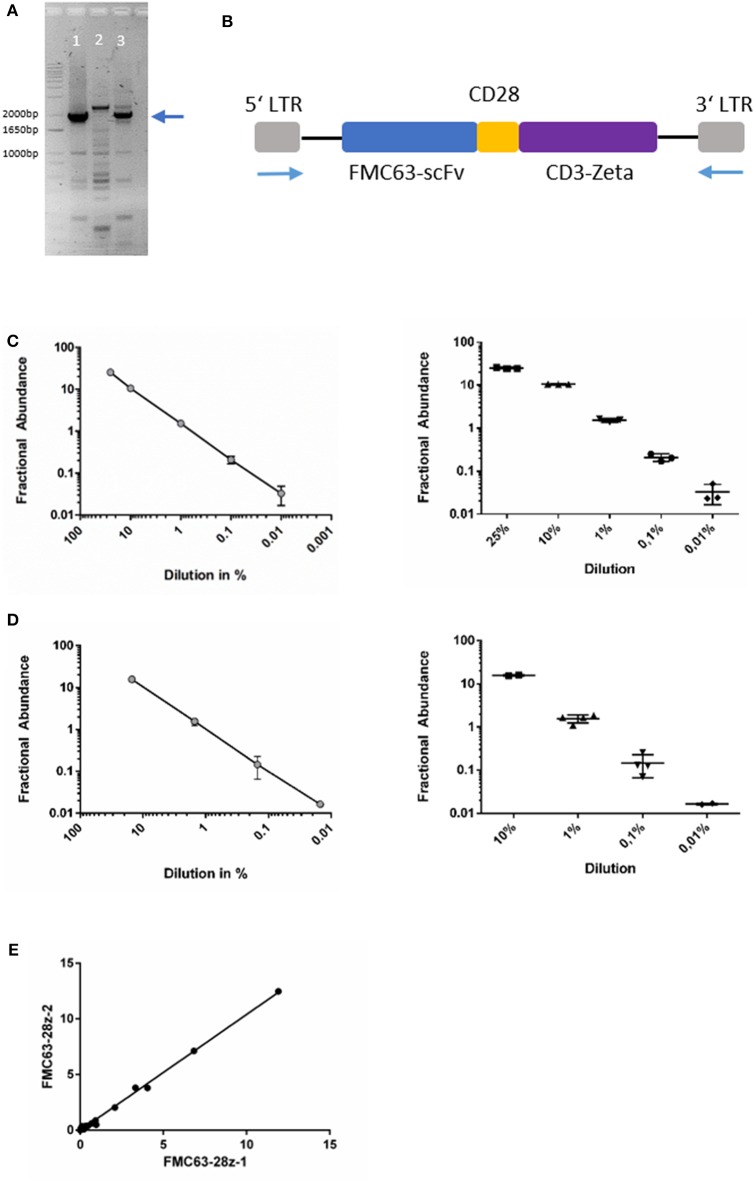
**(A)** Agarose gel-electrophoresis of PCR (primers binding to the LTR). Lanes 1&3: amplicons of patient-derived cells after axi-cel treatment, lane 2: negative control from un-transduced cells. The blue arrow indicates the desired amplicon. **(B)** Schematic image of axi-cel gene cassette. Blue arrows indicate primer positions used for amplification and sequencing. **(C)** Dilution series of spike-in experiments with primer-probe assay FMC63-28z-1. Slope of log-regression curve = 0.936. Pearson correlation with expected values *r*^2^ = 0.99. **(D)** Dilution series of spike-in experiments with primer-probe assay FMC63-28z-2. Slope of log-regression = 1.003. Pearson correlation with expected values r^2^ = 1. **(E)** Correlation of both assay's fractional abundance for all 54 samples analyzed (*r*^2^ = 0.998, *p* < 0.001).

As outlined above, the CD19-directed CAR-gene was amplified in a PCR reaction with LTR-directed primers. The amplicon could be separated as a distinct band and depicted at the expected length between 1,800 and 2,000 bp (Figures 1A,B). Gel-purification of the PCR product with subsequent DNA sequencing confirmed the assumed sequence as previously published by Kochenderfer et al. (2010, 2012).

### Detection of CD19-CAR Gene Sequences by ddPCR

Both ddPCR primer-probe assays discriminated reliably between FAM and HEX labeled droplet events (Supplementary Figure 3). We found high reproducibility and precision of the assay with standard deviations (SD) of 0.008 (FMC63-28Z-1) and 0.005 (FMC63-28Z-2) in replicate tests (*n* = 5) of positive control samples, respectively. In replicate tests of negative samples (*n* = 5), SD were 0 (FMC63-28Z-1) and 0.0009 (FMC63-28Z-2), respectively (Supplementary Figure 4). In 10 samples of patients not treated with axi-cel, no CAR-T-signal was found (*data not shown*). In summary, very low levels of CAR-T-signal could be quantified, with limit of blank (LOB) of 0 (FMC63-28z-1) and 2 (FMC63-28z-2) false positive droplets per assay, respectively.

To proof the feasibility of the designed ddPCR assay, we performed multiple spike-in PCRs comprising various concentrations of the gene of interest spiked into gDNA derived from un-transduced human T-cells. Dilution series demonstrated reliable detection of the CAR-gene in multiple replicates of probe with ~1 copy per genome (Figures 1C,D, Supplementary Figures 4A,B). The CAR sequence was consistently detectable in samples diluted to 0.01% in spike-in experiments with recovery ranging from 100 to 330% and 97 to 106%, respectively (Supplementary Table 1). The 0.01% dilution comprised 1.5 pg of the positive control DNA. That equals 0.25 diploid CAR-T-cell genomes (≈ 6 pg DNA per diploid genome). As 15 ng comprise the genomes of ~2,500 cells, our assay results in a limit of detection of 1 CAR-T-cell in a background of 10,000 cells. Because one CAR-T-cells may harbor more than one CAR-copy per genome, transduction efficacy (mean CAR-copies per genome) influences the limit of detection (Details of the statistical analyses are given in the Supplementary Data; Supplementary Figure 4, Supplementary Table 1). Correlation analysis between both ddPCR assays was carried out in 54 separate samples and resulted in r^2^ = 0.998, *p* < 0.001 (Pearson correlation, *t*-test; Figure 1E).

### CD19-CAR Detection in Patients Treated With Axi-cel

Next, we applied our ddPCR assay to quantify CD19-CAR in PBMCs obtained from seven patients treated with axi-cel over time. Each sample was analyzed with both assays (duplicates for each assay). Follow-up periods ranged between 4 weeks and 9 months. Patients' characteristics are outlined in Table 1.

**Figure 2:**
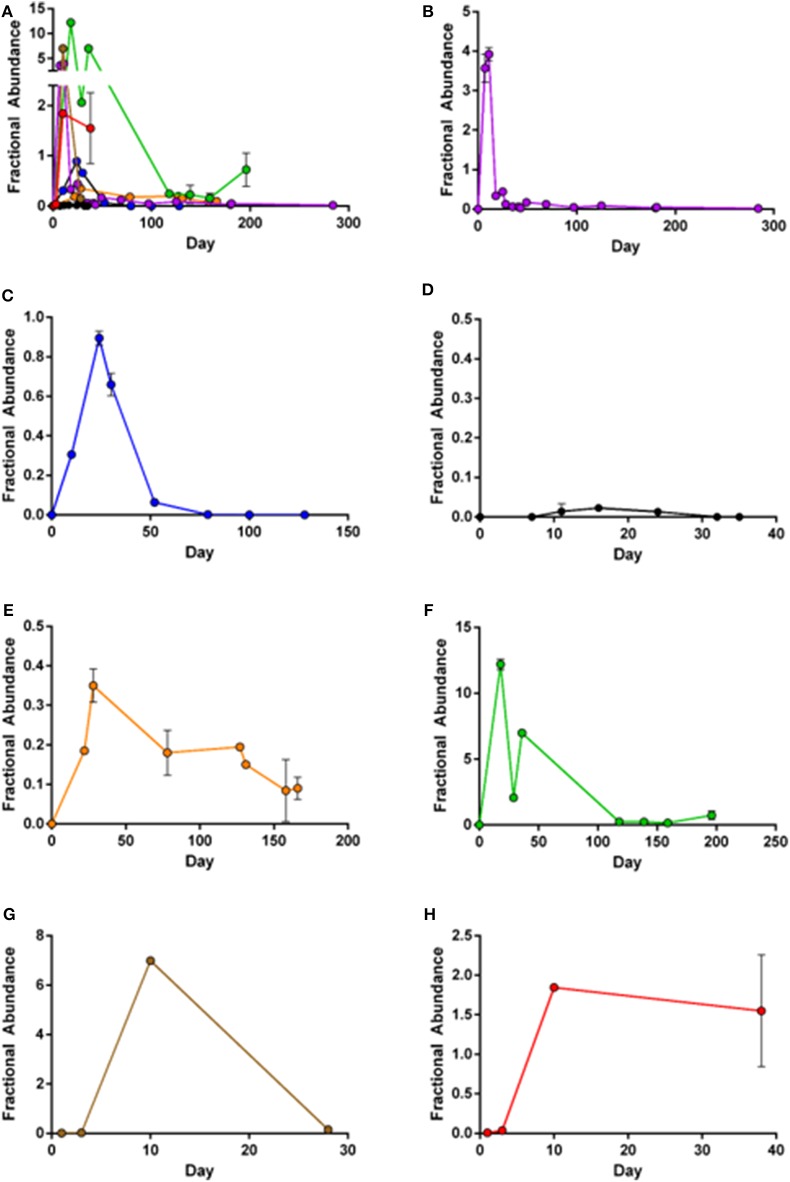
Mean fractional abundance of axi-cel (copies/μl) and housekeeper genes. Error bars indicate SD, showing high concordance of the two assays. **(A)** Merged image of all patient samples. **(B)** Patient 1: Peak expansion occurs during first weeks after infusion, but CD19-CAR are still detectable after 9 months follow-up. **(C)** Patient 2: Peak expansion followed by complete loss of CD19-CAR. **(D)** Patient 3: Limited detection of CD19-CAR, with low expansion and quick loss of CAR-T-cells. **(E)** Patient 4: Low expansion, but still unequivocal detection of CD19-CAR 160 days after infusion. **(F)** Patient 5: High expansion during first weeks after infusion with ongoing persistence. **(G,H)** Patient 6+7: Expansion during the 5 weeks after transfusion of patients recently treated.

Although CD19-CAR could be detected in PBMC from all axi-cel treated patients, significant differences in CD19-CAR expansion patterns were observed (Figure 2). In patients 1, 2, 4, and 5 (Figures 2B,C,E,F) initial CD19-CAR expansion was followed by decreasing numbers of copies. In PBMC from patient 2, no axi-cel copies could be detected at day 75 after re-transfusion. On the contrary, in PBMC of patients 1, 4, and 5 CD19-CAR copies were still detectable up to 9 months after re-transfusion. Notably, lymphoma remissions were observed in these patients, whereas patient 2 did not show lymphoma shrinkage during the 3 months follow-up period.

The lymphoma in patient 3 (Figure 2D) was stable in size after axi-cel treatment, and, CD19-CAR copies remained very low in sequential blood samples. Peak-expansion at very low copy levels was around day 20 and was followed by loss of CD19-CAR.

Patient 5 (Figure 2F) displayed the highest CD19-CAR copy levels. This measurable CAR-T cell expansion was in accordance with clinically complete remission (CR) during the 3 months follow-up period. Notably, patient 5 developed severe side effects following axi-cel therapy [CRS grade 2, ICANS grade 3 (Table 1)]. Limited follow-up of patients 6 and 7 (Figures 2G,H) shows increasing CAR-T-cells during the first weeks after transfusion. Both patients have not reached 3 months follow-up period.

Overall, peak expansion correlated with grade of ICANS (*r*^2^ = 0.58, *p* = 0.04) but not with CRS. All patients with loss of CAR-signal had disease progression.

### Confirmation of CAR-T-cell Detection by Flow Cytometry (FCM)

**Figure 3:**
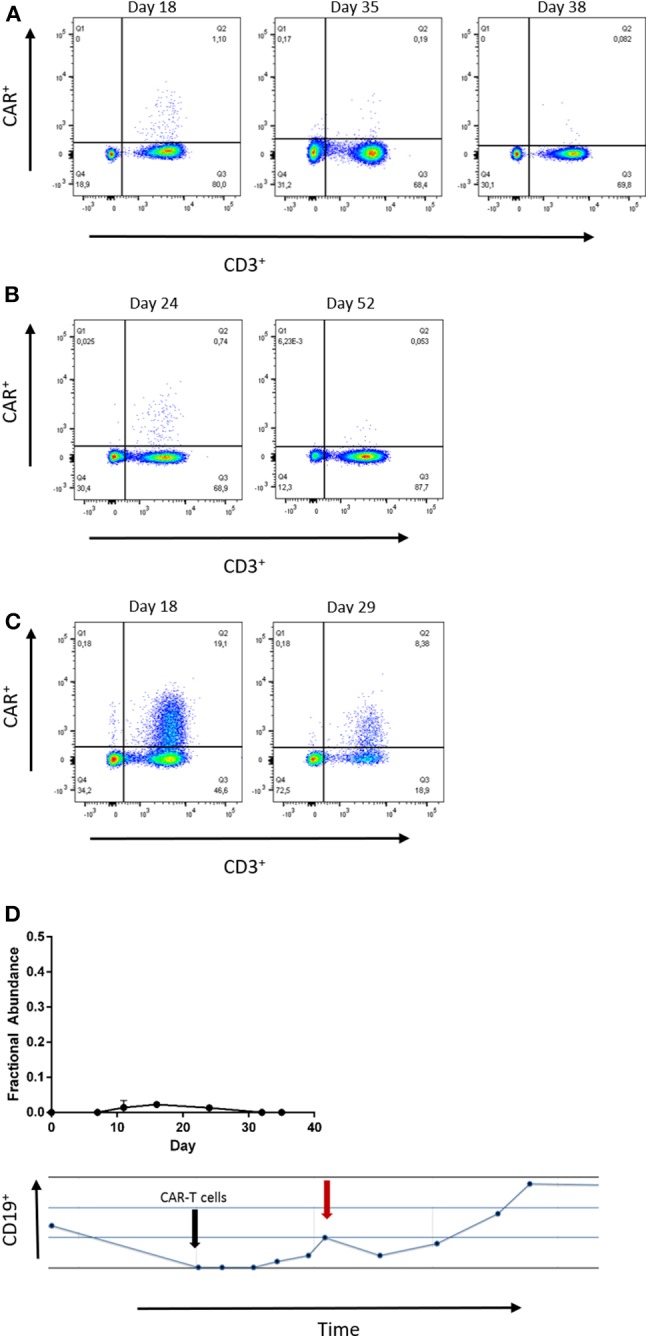
**(A–C)** Flow cytometry at the indicated time points of patient 1, 2, and 5. The number of CAR-T-cells detected by both, ddPCR and flow cytometry. Gating strategies included Lymphocytes by SSC/FSC gate and CD3 staining (APC). CD19-CAR detection kit was used with FITC-VioBright anti-Biotin antibody (Miltenyi Biotec™). **(D)** Low grade CD19-CAR detection in patient 3 is associated with reduction of absolute B-cell count. The black arrow indicates date of infusion, the red arrow indicates the time point of peak expansion.

Next, we confirmed the results of our ddPCR assay in FCM samples measured in parallel for patients 1, 2, and 5. Both, FCM and ddPCR showed decreasing amounts of CAR-T-cells over time (Figures 3A–C). Statistical comparison of ddPCR assays and FCM revealed a correlation of both methods (*r*^2^ = 0.929, *p* = 0.0005; Supplementary Figure 5, Supplementary Table 2).

Although CAR-T-cells were hardly detectable in blood samples of patient 3, we noticed decreasing B-lymphocyte counts at those time points when any CD19-CAR copies were detectable. This finding supports the reliability of detection of low CD19-CAR numbers in patient 3.

## Discussion

Quantification of CAR-T-cells in peripheral blood samples has exclusively been based on real-time quantitative PCR (qPCR) and flow cytometry (FCM). Currently, for both clinically approved CAR-T-cell products, tisagenlecleucel and axicabtagene ciloleucel (axi-cel), CAR-T cells have only been quantified within clinical trials. PCR assays for routine clinical diagnostics are not available. In this study, we have developed a digital-droplet PCR (ddPCR) assay for the CD19-CAR axi-cel and proved the feasibility of this test to reliably quantify CD19-directed CAR-T cells.

Preceding clinical trials have confirmed the strength of ddPCR to detect low-levels of target sequences in various applications, such as micro-chimerism analysis, minimal-residual disease (MRD) detection in hematologic malignancies, and liquid-biopsy (Klein-Scory et al., 2018; Link-Lenczowska et al., 2018; Mika et al., 2019; Franke et al., 2020). Real-time qPCR is technically challenging and complex, since qPCR depends on calculation of copies/μgDNA via standard-curves. This is avoided in our approach to quantify CD19-CARs by means of ddPCR. Here, we have used two primer-probe pairs and pooled the results from both assays, considering our previous results demonstrating increased sensitivity and retest-reliability of a ddPCR approach in chimerism analyses following hematopoietic stem cell transplantation (Mika et al., 2019).

Our ddPCR assay has technical overlaps with qPCR, yet, important differences make ddPCR a promising method for gene quantification in clinical routine and research settings. Both, qPCR and ddPCR quantify low amounts of gene copies in complex samples with high reproducibility and sensitivity (Della Starza et al., 2019; Pott et al., 2019). Furthermore, comparative studies have emphasized the ability of ddPCR to detect low amounts of copy numbers with high inter-laboratory reproducibility in MRD detection (Link-Lenczowska et al., 2018; Della Starza et al., 2019). We are confident that ddPCR represents a reliable and rapid tool for quantitative evaluation of CAR-T-cells.

False positive signals were ruled out by multiple testing of negative controls and performing dedicated spike-in analyses, which convinced us that even very low amounts of CD19-CAR gene copies can be specifically detected with our novel ddPCR assay. The assay performed with high precision, and, limit of 1/10,000 cells could be detected (LOD). Although assay FMC63-28z-1 overestimated CAR-T signals in low dilution samples, no false positive results were detected. Concordant course of B-cell counts and CAR-signal in patient 3 strengthened the clinical utility of our assay even at low levels of CAR-signal. Nevertheless, we are aware that this is a first proof-of-principle study and that higher numbers of samples have to be analyzed in order to ultimately define the limits of detection and clinical utility.

Transferring the results of our ddPCR assay from copies/μg gDNA into absolute counts of CAR-T cells is challenging, because cellular transduction efficacy in the final CAR product would have to be considered in each individual. Fractional abundance indicates the fraction of CD19-CAR genes as compared to diploid housekeeping genes. Thus, fractional abundance indicates relative amounts of CAR-genes per cell and could serve as surrogate parameter.

In the here analyzed patients, CAR-persistence and peak expansion were associated with clinical responses and ICANS, but not with CRS as previously reported (Neelapu et al., 2017). The ability of fractional abundance, e.g., as absolute threshold of peak expansion, to predict response or adverse events, has to be investigated in future studies, as the limited sample size is a limitation of the study.

A recently introduced commercial kit was used to verify the findings of our novel ddPCR assay by FCM. FCM has several limitations compared to PCR based assays, as has been detailed for MRD monitoring. Imponderabilities due to antibody and reagent variations, gating aspects, and deviations in cytometer calibration are potential confounders of inter-laboratory and retest-reliability (Garand et al., 2013). However, we observed significant correlation between the fraction of CD3^+^/CAR^+^-cells as measured by FCM and fractional abundance as detected by ddPCR. Due to limited sample size, these results have to be interpreted with caution, and, multiple confounders (e.g., transcription rate, transduction efficacy) have to be considered. Nonetheless, both approaches gave the same clinical message, meaning increasing or decreasing CAR-T-cell fractions in sequential blood samples from individual patients. This conformity between both methods strengthens the reliability of our ddPCR tests, yet, detailed comparisons in future studies comprising larger cohorts are required.

To summarize, our study is the first describing a ddPCR approach for detection and relative quantification of CD19-directed CAR-T cells following treatment with axi-cel in lymphoma patients. Although ddPCR remains a relatively new technique, we are certain that this method will find its place in routine clinical diagnostics and also clinical research to further follow and optimize these novel treatment approaches.

## Data Availability Statement

Publicly available datasets were analyzed in this study. This data can be found here: dHsaCP2500351, dHsaCNS674780718 (Supplementary Figure 2).

## Ethics Statement

The studies involving human participants were reviewed and approved by the Ethikkommission RUB. The patients/participants provided their written informed consent to participate in this study.

## Informed Consent

All patients agreed on publication of this study and any potentially identifiable data included and gave their written informed consent.

## Author Contributions

TM, AM, SK-S, SL-B, AB, JT, and JH collected patient samples and performed the experiments. TM, SH, GW, and RS designed the study and analyzed the data. TM and RS wrote the manuscript.

## Conflict of Interest

The authors declare that the research was conducted in the absence of any commercial or financial relationships that could be construed as a potential conflict of interest.
