# Supplementary material for: Digital-Droplet PCR for Quantification of CD19-Directed CAR T-Cells
Source: Front Mol Biosci. 2020 May 15;7:84. doi: 10.3389/fmolb.2020.00084 (PMC7243121; doi:10.3389/fmolb.2020.00084)
Supplement: Supplementary file 1 [file Data_Sheet_1.docx]

**Supplementary Data**

***Supp. Fig. S1A***

**Alignment of sequencing data:**


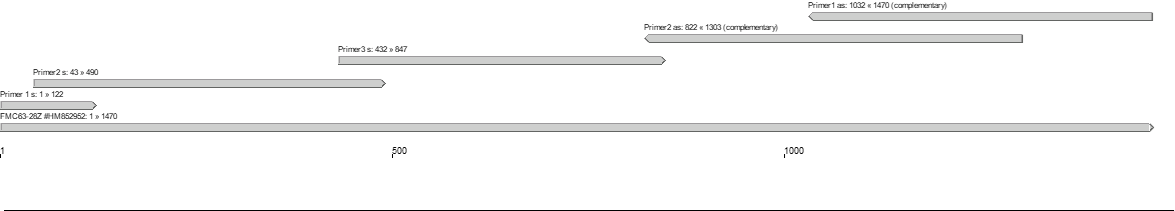


**Primers used for sequencing:** Primer 1 s: 5’ CAGCCCTCACTCCTTCTCTAG 3’

Primer 2 s: 5’ TGACAAGCCTTCTGCTCTGT 3’

Primer 3 s: 5’ GGAAATAACAGGCTCCACCT 3’

Primer 1 as: 5’ GCTAGCTTGCCAAACCTACA 3’

Primer 2 as: 5’ CCTCCGCCATCTTATCTTTC 3’

***Supp. Fig. S1B***

**Primers and probes used for 2 different ddPCR assays:**

Primer-Probe-Assay **FMC63-28Z-1**:

F 5’ TGGAAATAACAGGCTCCACC 3’

R 5’ CAGCTTACACCATAGTCGGG 3’

P FAM 5’ TCCACCAAGGGCGAGGTGAAACTGCA 3’ MGB

Primer-Probe-Assay **FMC63-28Z-2**:

F 5’ AACAGGGTAATACGCTTCCG 3’

R 5’ CCAGCTTACACCATAGTCGG 3’

P FAM 5’ ACCTGGCCTGGTGGCGCCCT 3’ MGB

**Figure S1: A** Alignment of the target sequence and all primers used for sequencing. **B** Sequences of primers and probes used for two different ddPCR assays for CAR quantification.

***Supp. Fig. S2***

**RPPH1 amplified sequence**

LOCUS X16612

DEFINITION Human gene for H1 RNA

ACCESSION X16612

VERSION X16612.1

5’ TTTTTTTCCAAAAATGGGCGGAGGAGAGTAGTCTGAATGGGTTATGAGGTCCCCTGCGG-GGTACCTCACCTCAGCCATTGAACTCACTTCGCTGGCCGTGAGTCTGTTCCAAGCTCCGGCAA 3’

**TERT amplified sequence**

LOCUS NR_149163

DEFINITION Homo sapiens telomerase reverse transcriptase (TERT),
 transcript variant 4, non-coding RNA

ACCESSION NR_149163

VERSION NR_149163.2

5’ CCAGACACTCTTCCGGTAGAAAAAGAGCCTGTTCTTTTGAAACGTGGTCTCCGTGACATAAAA-GAAAGACCTGAGCAGCTCGACGACGTACACACTCATCAGCCAGTGCAGGAACTTGGCCAG 3’

**Figure S2:** Nucleotide sequences of two reference genes (RPPH1 and TERT) which were used as control housekeeping genes. Unique assay identifiers (BioRad^TM^): dHsaCNS674780718 (RPPH1), dHsaCP2500351 (TERT).

***Supp. Fig. S3***

**
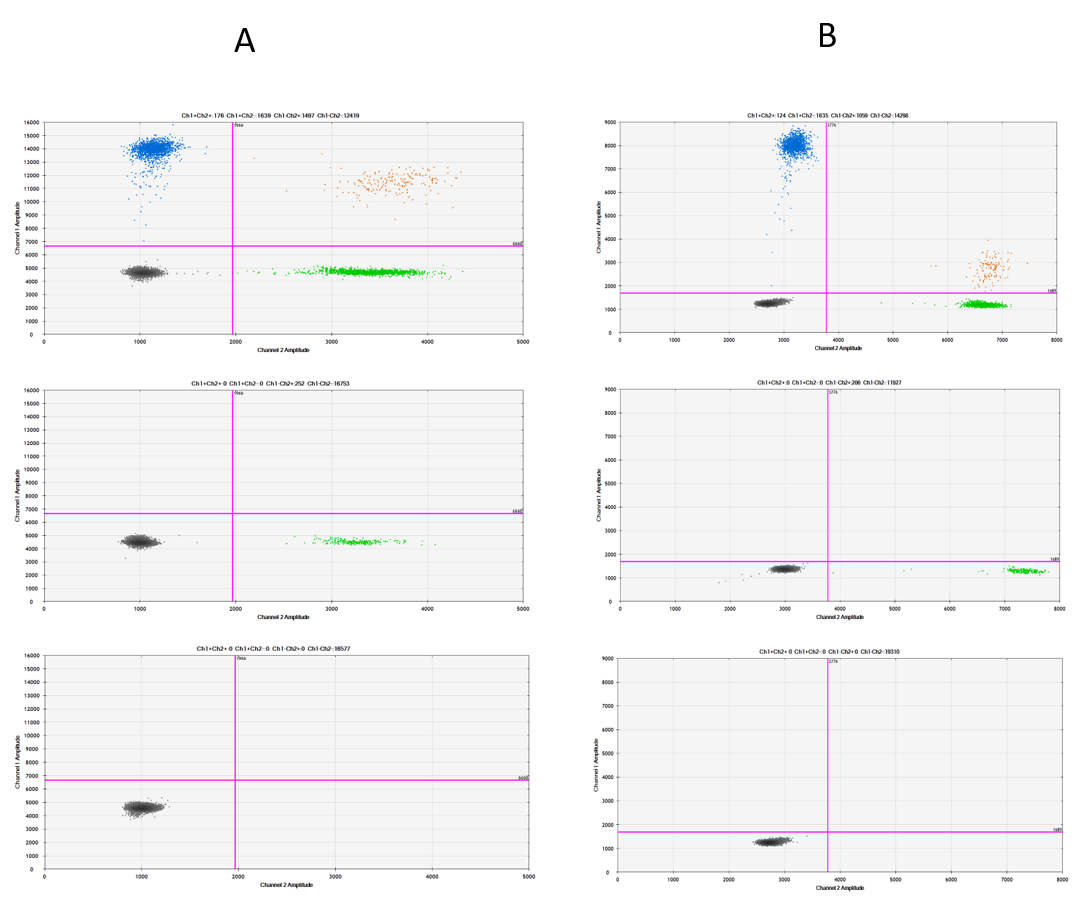
**

**Figure S3:** 2D-Amplitude of the assays performed. Blue: FAM positive calls, orange: double-positive counts, green: Hex positive counts, black: negative counts. **A** FMC63-28z-1 + RPPH1. Top: positive sample with CD19-CAR positive counts (blue + orange) and housekeepers (green). Middle: negative sample with positive housekeepers but no FAM counts. Bottom: Empty droplets. **B** FMC63-28z-2 + TERT. Top: positive sample with CD19-CAR positive counts (blue + orange) and housekeepers (green). Middle: negative sample with positive housekeepers but no FAM counts. Bottom: Empty droplets.

***Supp. Fig. S4***

***
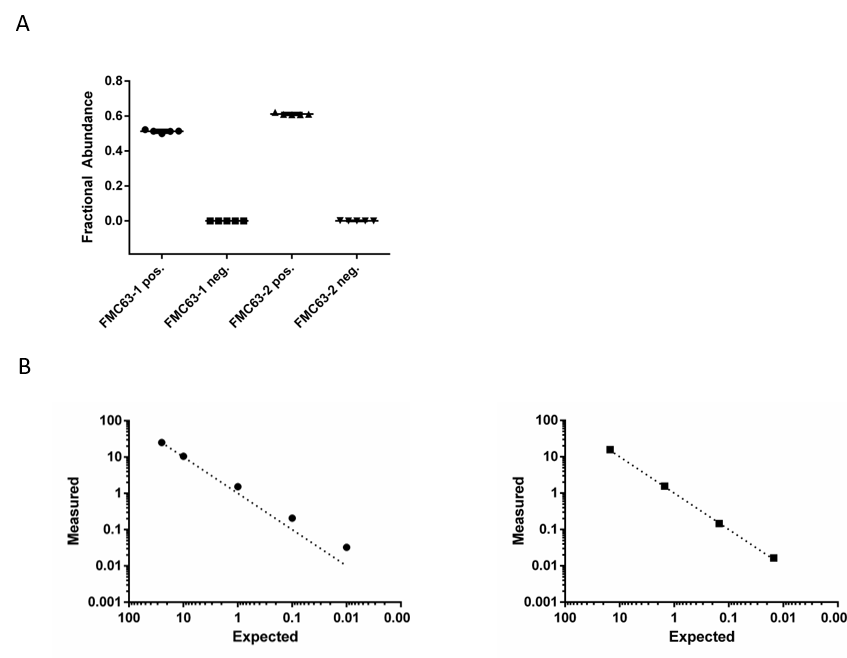
***

***Figure S4:*** **A** Replicate tests of positive control and negative control samples (each n=5). FMC63-28z-1: Pos. Ctrl.: Mean 0.51 [95%CI: 0.50-0.52], Neg. Ctrl.: Mean 0 [95%CI: 0.00-0.00]. FMC63-28z-2: Pos. Ctrl.: Mean 0.61 [95%CI: 0.61-0.62], Neg. Ctrl.: Mean 0.0004 [95%CI: -0.0007-0.0016]. **B:** Deviance from expected values (dotted line) for both assays in spike-in experiments. *Left:* *FMC63-28z-1+ RPPH1:* Increasing overestimation in lower dilutions. Mean squared error = 0.14. *right:* *FMC63-28z-2 + TERT:*  High accuracy in all dilution samples. Mean squared error = 0.11.

***Supp. Fig. S5***

***
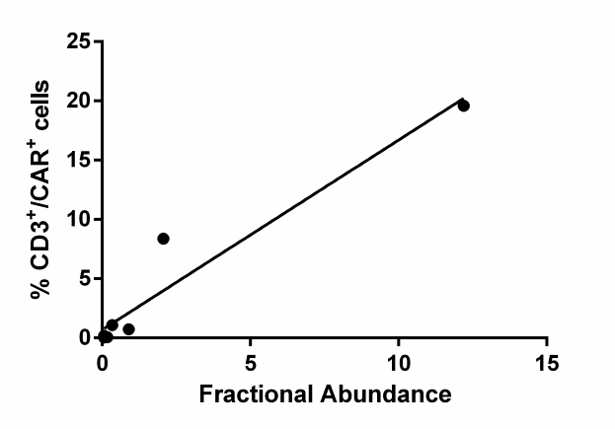
***

***Figure S5:*** Correlation of CAR-T cell in peripheral blood samples as assessed by flow cyto-metry (% of CD3^+^/CAR^+^ cells) and ddPCR (fractional abundance; n=7, r^2^=0,929, p=0.0005; **Supp. Tab. S2**).

***Supp. Tab. S1***

**FMC63-28Z-1**

| **Expected**  *(fractional abundance)* | **Measured**  *(mean)* | n | **SD** | **CV** (%) | **Recovery** (%) |
| --- | --- | --- | --- | --- | --- |
| 25 | 25.135 | 3 | 0.98 | 3.9 | 100.54 |
| 10 | 10.522 | 3 | 0.226 | 2.15 | 105.22 |
| 1 | 1.531 | 3 | 0.134 | 8.75 | 153.10 |
| 0.1 | 0.209 | 3 | 0.041 | 19.62 | 209.00 |
| 0.01 | 0.033 | 3 | 0.016 | 48.48 | 330.00 |

**FMC63-28Z-2**

| **Expected**  *(fractional abundance)* | **Measured**  *(mean)* | n | **SD** | **CV** (%) | **Recovery** (%) |
| --- | --- | --- | --- | --- | --- |
| 15 | 15.75 | 4 | 0.495 | 3.14 | 105.00 |
| 1.5 | 1.565 | 4 | 0.326 | 20.83 | 104.33 |
| 0.15 | 0.146 | 4 | 0.08 | 54.79 | 97.33 |
| 0.015 | 0.016 | 4 | 0.0007 | 4.375 | 106.67 |

***Table S1:*** Analytical performance of two ddPCR assays based on dilution series of positive controls. **FMC63-28Z-1:** Measured fractional abundance (mean from 3 replicate tests) shows increasing overestimation of CAR-signal in low-dilution samples. CAR-Signal is detectable in very low amounts. **FMC63-28Z-2:** Measured fractional abundance (mean from 4 replicate tests) shows high accuracy of the assay even in low-dilution samples.

***Supp. Tab. S2***

| ***patient / day*** | **ddPCR**  *(Fractional Abundance)* | **FCM**  *(% CD3+/CAR+ cells)* |
| --- | --- | --- |
| 1 / 18 | 0.335 | 1.1 |
| 1 / 35 | 0.059 | 0.19 |
| 1 / 38 | 0.17 | 0.082 |
| 2 / 24 | 0.895 | 0.74 |
| 2 / 52 | 0.064 | 0.053 |
| 5 / 18 | 12.195 | 19.6 |
| 5 / 29 | 2.065 | 8.38 |

***Table S2:*** Fractional abundance as assessed by ddPCR and corresponding results of flow cytometry (FCM) measurements in the same sample (n=7, p=0.0005, r^2^=0,929; **Supp. Fig. S5**).
